# Basal Cell Carcinoma Self-Treated With an Escharotic Agent (Black Salve) and Timeline of Cutaneous Responses

**DOI:** 10.7759/cureus.96519

**Published:** 2025-11-10

**Authors:** Gili Amid-Toby, Mario Sequeira

**Affiliations:** 1 School of Medicine, St. George’s University, Islip, USA; 2 Dermatology, Brevard Skin and Cancer Center, Rockledge, USA; 3 Dermatology, University of Miami/Jackson Memorial Hospital, Miami, USA

**Keywords:** alternative medicine, basal cell carcinoma, black salve, escharotic therapy, mohs surgery, sanguinarine, skin cancer treatment

## Abstract

Black Salve is an escharotic used in alternative medicine for the treatment of a variety of skin conditions, including benign growths and skin cancers. These products are readily available on the Internet, are unregulated, and are used by patients seeking non-surgical cures. We present a case study of a basal cell carcinoma (BCC) self-treated with this agent. The patient meticulously documented every step of treatment, providing invaluable information for medical professionals in understanding, counseling, and properly managing such clinical scenarios involving escharotic use.

## Introduction

Basal cell carcinoma (BCC) is the most common malignancy in humans, typically arising on sun-exposed areas of fair-skinned individuals [1]. Although it rarely metastasizes, BCC can cause significant local tissue destruction if not treated appropriately. First-line treatments include curettage and electrodesiccation, surgical excision, Mohs micrographic surgery, cryotherapy, and topical therapies, all of which have well-documented efficacy and safety profiles [2].

By contrast, some patients pursue unproven alternative therapies such as Black Salve, a topical escharotic compound typically containing zinc chloride, bloodroot (*Sanguinaria canadensis*), and other synthetic and botanical products. Bloodroot was once used by native Americans to treat cancer, and different formulations have been developed over the years, including Fell’s paste (1850s), Hoxsey’s red paste (1920s), and Mohs’ paste (1930s) [3]. Although Black Salve contains cytotoxic alkaloids like sanguinarine that have shown limited cancer cell toxicity in vitro, these effects are highly concentration-dependent and not reliably selective. At higher or variable doses, common in unregulated formulations, these agents damage both malignant and healthy tissue, leading to indiscriminate tissue necrosis and potentially disfiguring outcomes [3]. Despite warnings from the U.S. Food and Drug Administration (FDA) about its dangers, Black Salve continues to be marketed and used for self-treatment of skin cancer [4]. Dermatologists are often unfamiliar with the clinical phases of cutaneous response to the application of these products.

This report presents a patient who self-treated a biopsy-proven nodular BCC of the left nasal crease with Black Salve (Cansema^R^ Salve Deep Tissue, Alpha Omega Labs, Miami, FL) and photographically documents the complete treatment cycle from initial application through eschar formation, cavitation, re-epithelialization, and definitive Mohs surgical excision [5]. 

## Case presentation

**Figure 1:**
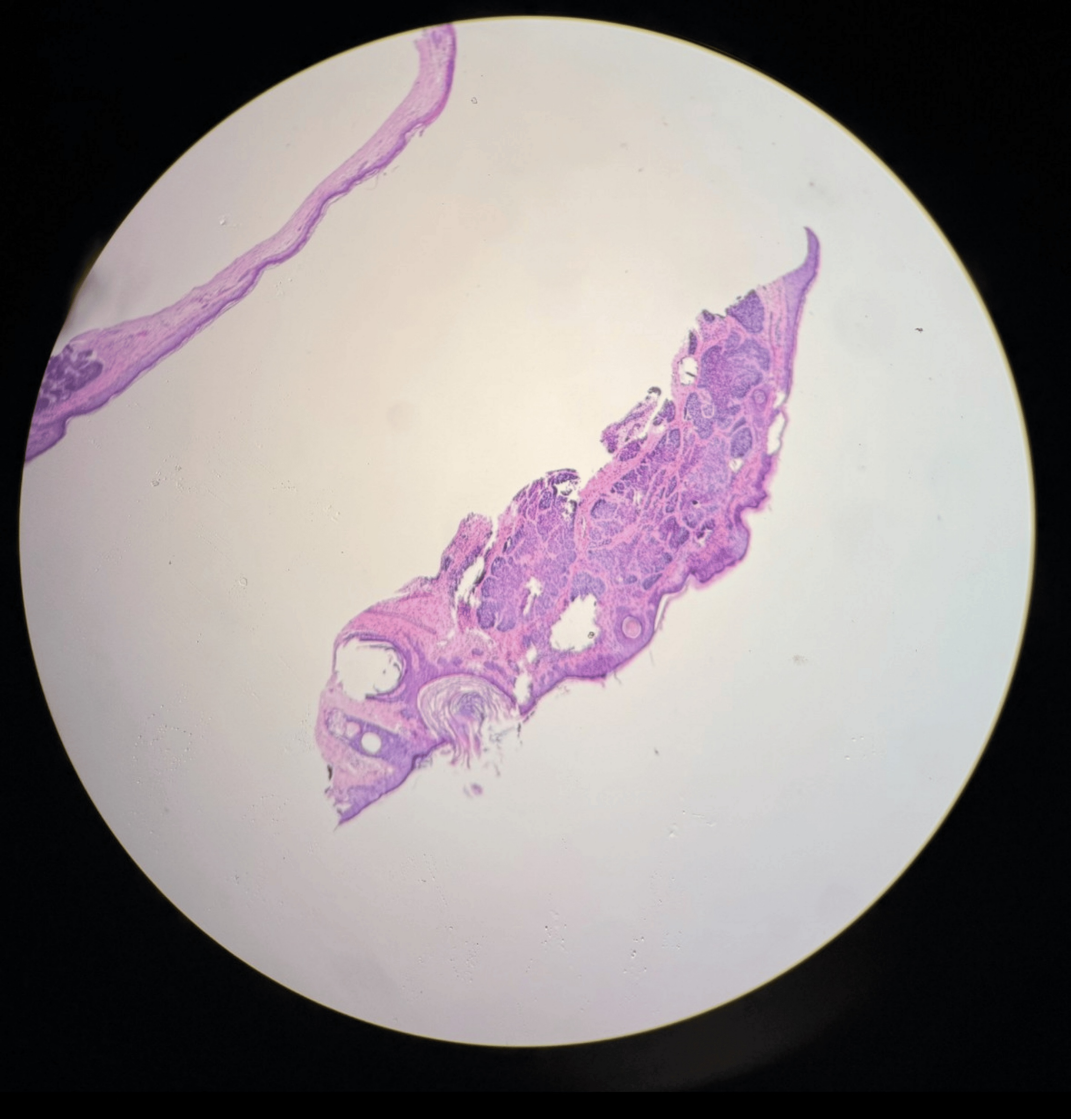
Histology of biopsy-proven nodular basal cell carcinoma (BCC).

A 68-year-old male with a history of chronic sun exposure and recent diagnosis of metastatic melanoma of the right axilla, about to start intravenous treatment with pembrolizumab every three weeks, presented for Mohs surgical consultation of a biopsy-proven nodular BCC of the left nasal crease measuring 2 cm (Figure 1).

**Figure 2:**
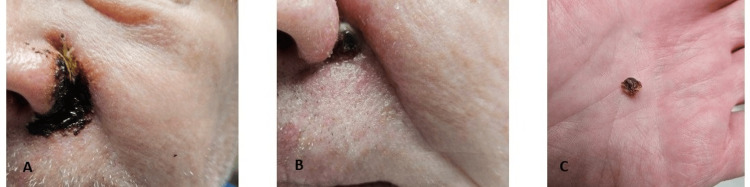
A. Application of Black Salve. B. Eschar formation. C. Sloughed eschar (day 9).

**Figure 3:**
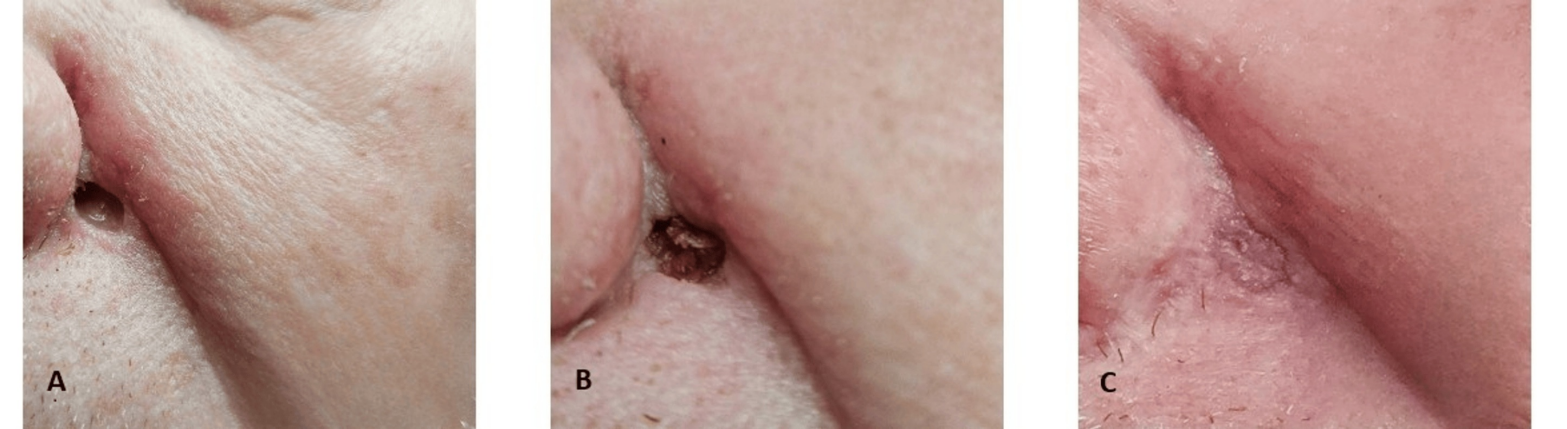
A. Cavitation or pit after eschar falls off (day 10). B. Ulcer healing by second intention (day 11). C. Healed wound (day 20).

The patient opted to use Black Salve to avoid surgery based on his erroneous belief that it was natural and described as anecdotally curative for skin cancer treatment on Internet sites [3]. In 2019, he self-treated a biopsy-proven superficial spreading malignant melanoma (0.8 mm, Clark level IV) on his back and never followed up for definitive surgery as he assumed the site had cleared upon completion of the Black Salve application cycle. On May 8, 2025, the patient self-administered the escharotic on his left nasal crease over the lesion and surrounding skin margin, creating a lightly caked appearance (Figure 2A). After 24 hours, any remaining product and organic material, such as crust and serous fluid, was carefully removed using a Q-Tip and 3% hydrogen peroxide. An eschar formed at the site, which eventually sloughed off on day nine post initial application, revealing an ulceration (Figure 2B, 2C, 3A).

This ulcer gradually re-epithelialized and fully healed by day 20 (Figure 3B, 3C).

**Figure 4:**
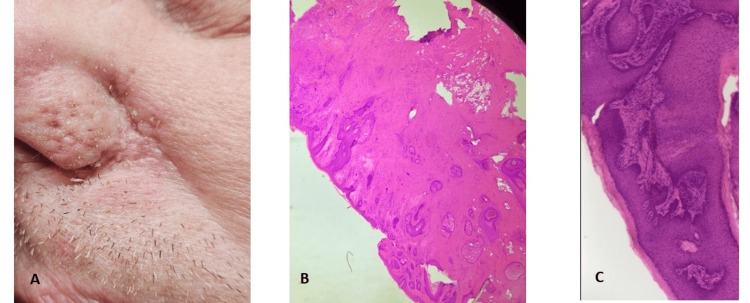
A. Scar following two cycles of Black Salve. B. H & E of Mohs first section. Scar and inflammatory response. C. H & E. Pseudoepitheliomatous hyperplasia and reactive inflammation.

The patient then reinitiated an abbreviated second application cycle with a reduced amount of Black Salve, resulting in a smaller eschar and ulceration, which fully healed by the tenth day, two days prior to his Mohs surgical appointment (Figure 4A). His procedure was uneventful, requiring one Mohs stage to achieve clear margins. Histologically, lymphocytic inflammation, pseudoepitheliomatous hyperplasia, and scarring were observed (Figure 4B, 4C).

**Figure 5:**
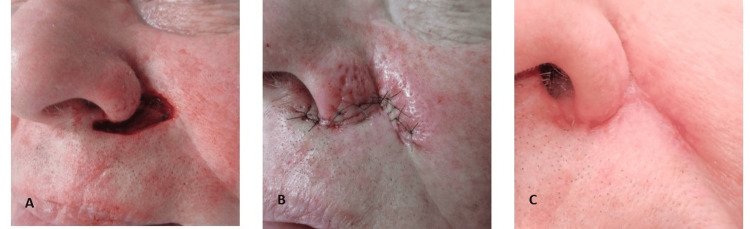
A. Mohs surgical defect. B. Single advancement flap repair. C. Postoperative results at seven weeks.

Deeper cuts in the tissue block, as well as a superficial epidermal layer of the original biopsy site, failed to reveal residual BCC. The resulting defect was repaired with a single advancement flap, and the patient recovered free of complications and with excellent cosmesis (Figure 5A, 5B, 5C).

## Discussion

Black Salve is a topical escharotic compound commonly marketed as a natural herbal treatment for skin cancer. Its main ingredients, zinc chloride and sanguinarine, induce coagulative necrosis, leading to eschar formation and eventual tissue sloughing [3]. Although anecdotal reports suggest it can eliminate lesions, Black Salve remains unregulated, and its clinical efficacy and tissue selectivity have not been validated in controlled studies [6]. In fact, the literature documents severe adverse outcomes associated with its use, including scarring, abnormal pigmentation, and disfigurement when used on the nasal ala [3].

Our case is unique in that it documents every step of the patient’s self-treatment cycle and sheds valuable information on this alternative product. The timeline started with the application of Black Salve, during which patients can experience tingling or burning lasting from minutes to hours, sometimes requiring oral analgesics. After 24 hours, the site was cleansed with 3% hydrogen peroxide to manage the developing eschar. In cases where serous fluid or exudate is minimal, a second dose of the escharotic agent may be needed with large lesions requiring up to three applications. The dressing is replaced and the site cleansed according to the level of discharge. Removal of the eschar follows, which typically happens naturally within 10 days and should not be forced prematurely to prevent scarring. Cavitation, or the formation of a pit or ulcer after the eschar falls, is next, leading to second intention healing during which infection prevention is crucial. Additional escharotic treatment cycles (second or third) are not advisable to minimize the risk of scarring.

In our patient, Mohs surgery confirmed no residual BCC histologically after one stage. While this could reflect spontaneous tumor regression or prior biopsy effect, this outcome should be interpreted with caution, as the sanguinarine alkaloid could have indiscriminately worked by destroying cancerous and non-cancerous tissues alike [3]. Use of the product in the absence of highly effective confirmatory excisional or Mohs surgical treatment should be emphatically discouraged, as there is no long-term follow-up data on the efficacy of Black Salve, but rather, reports of recurrences, metastasis, and death because of its use [3]. Our patient is currently grappling with metastatic melanoma, presumably due to prior use of the product six years ago.

Black Salve-treated skin shows the two main histological patterns of dermal scarring (with or without a granulomatous reaction, foreign material, or stromal atypia) and ulceration with suppurative inflammation and tissue necrosis [7,8]. These features can obscure residual tumor or mimic malignancy, complicating diagnosis. Tumor skip areas have also been reported at the time of Mohs surgery [9]. While some patients experience visible lesion regression, it must be emphasized that others have had treatment delays, disfiguring scarring, or progression to advanced disease, including metastasis in both BCC- and melanoma-treated lesions [6,10]. In our case, the presence of scarring, reactive inflammation, and pseudoepitheliomatous hyperplasia following healing of the ulceration made interpreting histologic Mohs sections more difficult.

## Conclusions

This report documents a full treatment cycle with Black Salve and uniquely helps medical practitioners better manage and counsel patients who are acquiring and using these products on their own to avoid surgery. The field of alternative medicine is broad, and practitioners are often faced with a knowledge gap in addressing complications arising from the use of such readily available products. As practitioners, we must educate self-treating patients on the potential serious complications associated with Black Salve use, encourage definitive surgery, and offer close long-term follow-up to those patients who refuse. In this article, we describe four distinct phases associated with Black Salve use, including application, eschar management, cavitation, and second intention healing. We also warn of challenges created by this escharotic in interpreting Mohs histologic sections.

Further well-controlled clinical trials with consistent bloodroot alkaloid formulations are needed to determine the role of Black Salve in the treatment of skin conditions, including skin cancer, as well as long-term follow-up on recurrence data. For the time being, Black Salve is unsuitable as a substitute for evidence-based cancer therapies.
